# Effects of Rare Earth Elements on the Isothermal Oxidation of the Alumina-Scale-Forming NbSiTiAlHf Alloys

**DOI:** 10.3390/ma18225182

**Published:** 2025-11-14

**Authors:** Chang Jiang, Hui Zhao, Dan Wu, Song Zeng, Youxing He, Xuebing Yang, Linwei Zhang, Jiuming Yu, Lei Lu, Wenfu Chen

**Affiliations:** 1 Jiangxi Key Laboratory of Advanced Copper-Based Materials, Institute of Materials and Intelligent Manufacturing, Jiangxi Academy of Sciences, Nanchang 330096, Chinaxbyjz@163.com (X.Y.); llullei@163.com (L.L.); 2 School of Physics and Materials Science, Nanchang University, Nanchang 330031, China

**Keywords:** NbSiTiAlHf alloys, rare earth elements, the isothermal oxidation, the alumina scale, spallation

## Abstract

The microstructures and oxidation behavior of the NbSiTiAlHf alloys doped with rare earth elements at 1300 °C were investigated. The nominal compositions of the selected alloys are Nb-13.5Si-23Ti-37Al-5Hf-0.5X (at.%), where X = Y, Dy, and La, respectively. It was shown that the whole scales were mainly composed of the major phases of Al_2_O_3_ and the minor phases of TiO_2_, where the TiO_2_ formed on the surface or in the upper layer of scales, for the undoped, Y, and Dy-doped alloy. But, for the 0.5 at.% La-doped alloys, the whole scales were constituted with the major phases of both Al_2_O_3_ and TiO_2_, and contained plenty of large voids. The 0.5 at.% Dy-doped alloys exhibited the lowest scale growth rate with the value of 1.87 × 10^−11^ cm^2^/s, and the benefits of Y on the oxidation rates were short-term, while 0.5 at.% La-doped alloys had the highest scale growth rate of 4.55 × 10^−10^ cm^2^/s compared with those of all the selected alloys. Then, the effects of Y, Dy, and La on the oxidation behavior of the alumina-scale-forming NbSiTiAlHf alloys were discussed.

## 1. Introduction

With the development of aerospace technology, higher thrust-to-weight ratios and work efficiency are required for aircraft engines and other propulsion systems, thus placing higher demands on high-temperature structural materials used in aircraft engines. The widely used high-temperature structural material for engines at present is Ni-based single-crystal high-temperature alloys, which can operate at temperatures of up to 1150 °C as they approach their temperature limit (limited by the melting point of Ni) [1,2]. Therefore, there is an urgent need to develop a new generation of high-temperature structural materials with enhanced high-temperature strength, superior creep resistance, good room-temperature fracture toughness, and excellent high-temperature oxidation resistance. In recent years, Nb-Si based superalloys, with a lower density of 6.6–7.2 g/cm^3^ than Ni based alloys, liquidus temperatures above 1900 °C, better high-temperature strength and room temperature fracture toughness, are expected to be used at temperatures between 1200 and 1400 °C or even higher, becoming one of the most potential candidate materials to replace Ni based superalloys for aircraft engines [3,4].

In the practical application of high-temperature structural materials, oxidation resistance usually controls the service life of the materials. Unlike Ni-based superalloys, the Nb-Si-based superalloys are prone to pesting oxidation at high temperatures, which form an oxide layer consisting of titanium niobate, AlNbO_4_, CrNbO_4_, and oxides of Nb and Ti with a loose structure, failing to generate a dense and protective oxide film. This results in poor high-temperature oxidation resistance, limiting their development and application [5,6]. Despite this, extensive research has shown that alloying with elements such as Cr, Al, Ti, B, Ge, and Hf can improve the high-temperature oxidation resistance of Nb-Si-based alloys to a certain extent. However, these elements often degrade the mechanical properties, such as room-temperature fracture toughness, high-temperature strength, or creep resistance [7,8,9]. Therefore, the coating technology is a more effective method to enhance the oxidation resistance of Nb-Si-based ultra-high temperature alloys.

To ensure that the protective coatings for Nb-Si-based alloys exhibit excellent oxidation resistance, apart from requiring a dense coating structure, the following essential requirements should be satisfied in material selection and compositional design: (1) low growth rate of the protective oxide layer; (2) crack resistance, with a thermal expansion coefficient close to that of the alloy substrate to avoid cracking or peeling during service; (3) low volatility of the other coating constituents and the protective oxide layer; and (4) minimal elemental interdiffusion between the coating and the alloy substrate during high-temperature oxidation [9]. At present, the protective coatings of Nb-Si-based alloy are mainly silicide coating systems, demonstrating good high-temperature oxidation resistance [10,11,12,13]. However, composite silicide coatings suffer from serious interdiffusion with the niobium alloy substrate at high temperatures. Additionally, the oxide layer formed on the surface of silicide coatings at high temperature contains SiO_2_, which volatilizes significantly above 1650 °C. These factors will lead to premature failure of the coating and reduce the service life of Nb-Si-based alloy in the high-temperature environment. Thus, alloy materials, which have good chemical compatibility with the substrate and form a dense Al_2_O_3_ layer at high temperatures, are ideal coating materials for Nb-Si-based alloys, since Al_2_O_3_ has higher thermodynamic stability than SiO_2_. An NbSiTiAlHf high-entropy alloy as coating material, which consists of (Ti,Nb)Al_3_, Ti_5_Si_4_, TiSi, and Nb_5_Si_3_, can form a continuous and dense Al_2_O_3_ layer on the surface after being oxidized at 1200 °C, demonstrating excellent high-temperature oxidation resistance. Moreover, the composition of this alloy is similar to that of the Nb-Si-based alloy substrate [14,15,16], making it a promising candidate for ultra-high-temperature coatings on Nb-Si-based alloys.

It is well known that rare earth elements (such as Y, Dy, etc.) can significantly improve the oxidation resistance of Ni-based superalloys or protective coatings (which form Al_2_O_3_ or Cr_2_O_3_ oxide layers at high temperatures) due to their unique physical properties. Their effects are primarily reflected in the change to the structure, growth, and adhesion of thermally grown oxide layers [17,18,19]. To enhance the high-temperature oxidation resistance of silicide protective coatings on Nb-Si-based alloys, research has shown that the addition of rare earth elements (such as Y, Ce, etc.) to silicide coatings can refine the coating microstructure, improve adhesion of the oxide layer, and thus enhance oxidation resistance [11,20,21]. Currently, the effect of rare earth elements on the high-temperature oxidation resistance of NbSiTiAlHf high-entropy alloys has not been investigated; the mechanisms by which rare earth elements influence the structure, growth, and adhesion of thermally grown oxide layers on NbSiTiAlHf high-entropy alloys are unclear. In this study, the microstructures and oxidation behavior of NbSiTiAlHf high-entropy alloys with different rare earth species were investigated, and their effect was discussed.

## 2. Materials and Methods

### 2.1. Sample Preparation

The NbSiTiAlHf alloys doped with rare earth elemental species (Y, Dy, and La) were prepared as small (30 g) buttons by arc-melting high purity elemental particles (99.9%, Naiou Nano, Shanghai, China) of niobium, titanium, silicon, aluminum, hafnium and trace rare earth elements (Y, Dy and La) in a water-cooled copper crucible, using a non-consumable tungsten electrode. Elemental particles were used with a rod or amorphous size of 2–3 mm. The particles were cleaned, degreased, and dried before being placed in a water-cooled copper crucible. The vacuum system was started after loading, and the argon was introduced when the vacuum was lower than 10^−3^ Pa. Then, the pre-placed titanium ingot was melted to further reduce the oxygen content in the furnace. During melting, the particles of Nb, Ti, and Hf, as well as the particles of Al and Si, were first melted into an intermediate ingot, respectively (to improve composition uniformity and reduce burning loss of the element with a low melting point). Then, the two intermediate ingots were melted together, and the final melting procedure was repeated 5 times for each alloy. Electromagnetic stirring was used in all of the smelting processes. The mole fractions of Nb, Si, Ti, Al, and Hf were 13.5%, 23.0%, 23.0%, 37.0% and 3.5% in all precast alloys, respectively. The mole fractions of the trace rare earth element added separately were 0.5%.

### 2.2. Thermal Treatment

The as-deposited specimens were annealed in vacuum (<10^−2^ Pa) at 1300 °C for 24 h to homogenize the composition, and then, the specimens were cut into small slices of 5 mm × 5 mm × 4 mm by the wire electrical discharge machining. The six sides of the slice specimens were polished to 1200 grit. The oxidation tests were performed in a box furnace with static air. All the alumina crucibles used for loading specimens were dried at 1300 °C for 2 h. Then the slice specimens placed in the furnace center were subjected to isothermal heat treatment at 1300 °C with holding times (h) of 12, 24, 48., 96, and 192.

### 2.3. Characterization Methods

The surface and cross-sectional microstructures of the alloys were investigated by a scanning electron microscope (SEM, Zeiss Gemini 300, Carl Zeiss, Oberkochen, Germany) coupled with an energy-dispersive spectrometer (EDS, AZtec X-Max 50, Oxford Instruments, Oxford, UK). The phases of the alloys and the oxidized specimens were detected using X-ray Diffraction (XRD, Ultima IV, Rigaku, Yamanashi, Japan) with a Cu-Kα radiation at a recorded 2θ range from 10° to 90° (40 kV and 30 mA).

The oxidation kinetics curves of the alloys are obtained by measuring the thermally grown oxide (TGO) thickness. The samples of each holding time period were polished after Ag/Ni plating protection treatment for the oxide layer on the surface, and then the section was ground. The TGO thickness is obtained by dividing the TGO area of each selected section by the length. At least four measurement sections are selected for the samples of each holding time period.

## 3. Results and Discussion

### 3.1. The Microstructures of the NbSiTiAlHf Alloys After Vacuum Annealing

**Figure 1:**
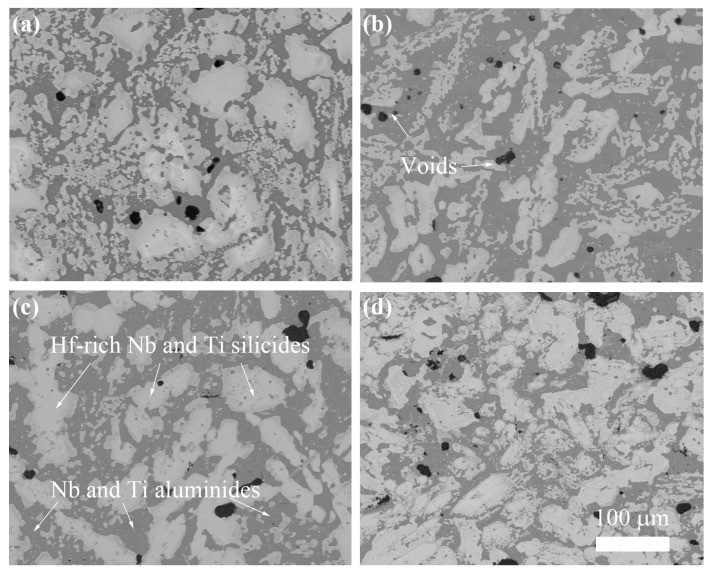
The cross-sectional images of the alloys after annealing in a vacuum at 1300 °C: (**a**) the NbSiTiAlHf alloys undoped; (**b**) doped with 0.5 at.% Y; (**c**) dopped with 0.5 at.% Dy; (**d**) doped with 0.5 at.% La.

**Figure 2:**
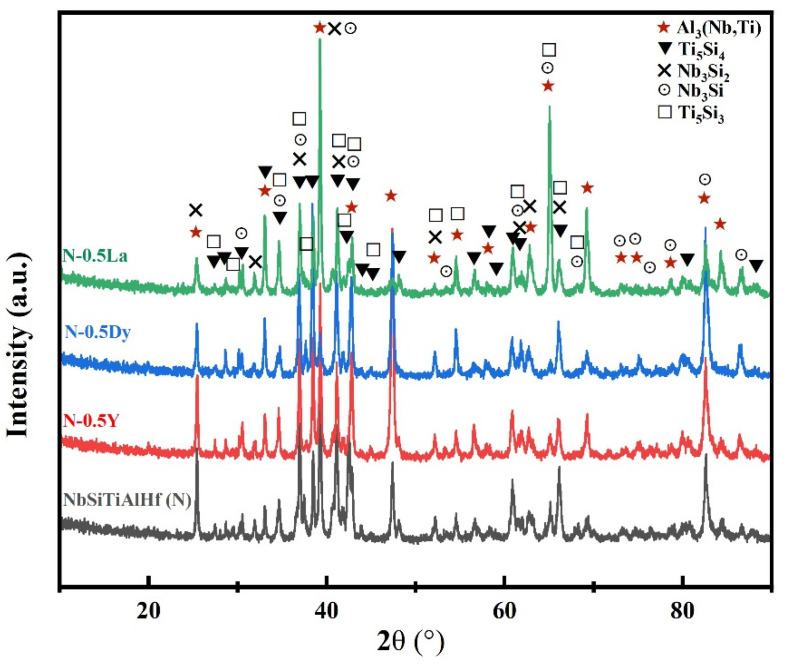
The XRD patterns of the NbSiTiAlHf alloys, undoped and doped with 0.5 at.% Y, 0.5 at.% Dy, and 0.5 at.% La, after annealing in a vacuum at 1300 °C.

Figure 1 shows the cross-sectional images of the NbSiTiAlHf alloys, undoped and doped with 0.5 at.% Y, 0.5 at.% Dy, and 0.5 at.% La, after annealing in vacuum at 1300 °C. From the EDS analysis, the bright areas of the undoped alloy could consist of Hf-rich Nb and Ti silicides, which are rich in the elements Nb, Si, Ti and Hf; meanwhile, the dark areas consist of Nb and Ti aluminides, which are rich in the elements Nb, Ti and Al, further confirmed by the XRD analysis (Figure 2). According to the XRD results, the phases of Nb and Ti silicides are mainly identified as Nb_3_Si_2_, Ti_5_Si_4_, Nb_3_Si, and Ti_5_Si_3_, and the phases of Nb and Ti aluminides are mainly identified as (Nb,Ti)Al_3_. The phases of the alloys doped with different rare earth elements are almost the same as the undoped specimens, owing to the low concentrations of the doped elements (0.5 at.%).

It can also be observed that all of the alloys still show microstructural and compositional inhomogeneity after being annealed in a vacuum at 1300 °C for 24 h, which is attributed to the coexistence of multiple elements in NbSiTiAlHf alloys. These elements exhibit intrinsic differences in melting points, vapor pressures, and other physical properties, which makes it challenging to control the composition and microstructures during melting [22,23]. Small amounts of voids can be observed in all of the alloys, probably due to the low flowability of the alloy melt. The silicides and aluminides are generally cross-distributed, with significant differences in the morphology of different phases. The inhomogeneous morphology is shown in all of the alloys, consistent with other observations [15].

### 3.2. The Microstructures of the NbSiTiAlHf Alloys After Oxidation

#### 3.2.1. The Surface Microstructure of the Oxide Scales

**Figure 3:**
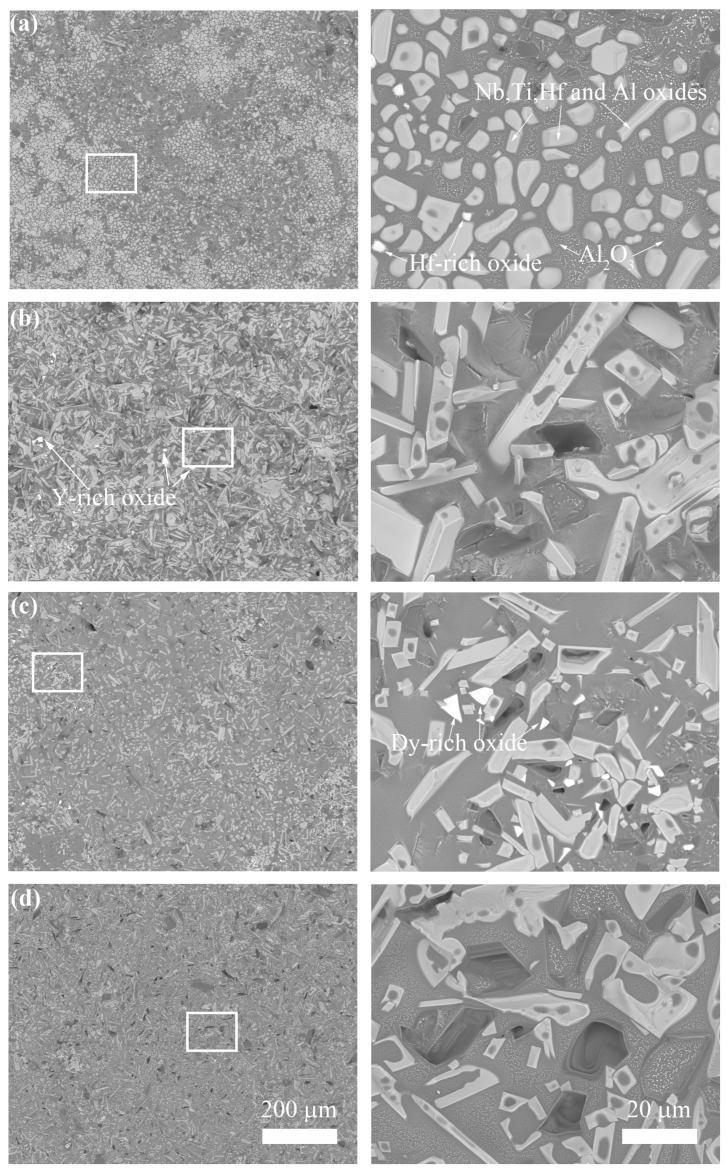
The surface morphologies of the NbSiTiAlHf alloys oxidized for 48 h at 1300 °C: (**a**) the undoped alloy; (**b**) doped with 0.5 at.% Y; (**c**) doped with 0.5 at.% Dy; and (**d**) doped with 0.5 at.% La.

**Figure 4:**
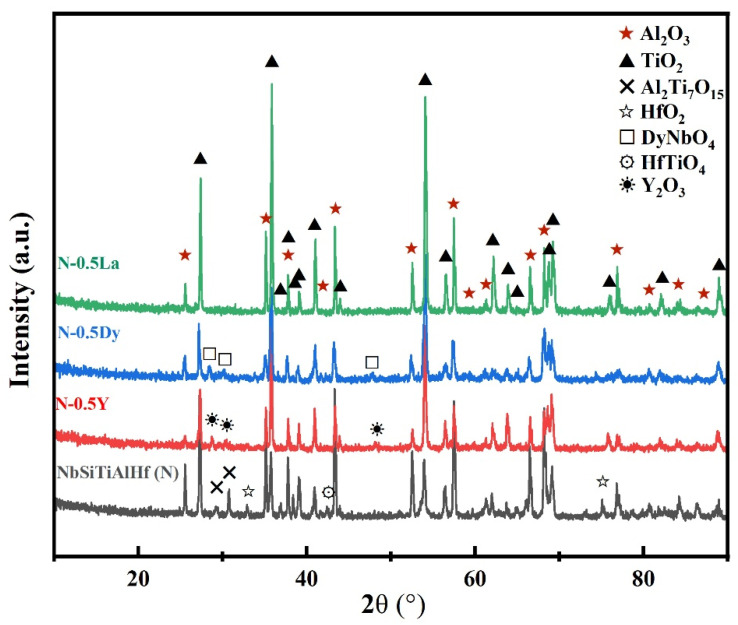
The XRD patterns of the NbSiTiAlHf alloys with and without rare earth elements oxidized for 48 h at 1300 °C.

Figure 3 shows the surface morphologies of the NbSiTiAlHf alloys with and without rare earth elements oxidized for 48 h at 1300 °C: (a) undoped, (b) doped with 0.5 at.% Y, (c) doped with 0.5 at.% Dy, and (d) doped with 0.5 at.% La. From the EDS analysis, the bright areas on the surface of the oxide scale for the undoped alloy could be made up of Hf-rich oxide, and composite oxides are rich with the elements of Nb, Ti, Al, and Hf; meanwhile, the dark areas are mainly Al-rich oxides (Figure 3a). Further, based on the XRD analysis (Figure 4), the Nb, Ti, Al, and Hf-rich composite oxides are confirmed as the major phase of TiO_2_, minor phases of Al_2_Ti_7_O_15_ and HfTiO_4_; Hf-rich oxides and Al-rich oxides are identified as HfO_2_ and Al_2_O_3_, respectively. On the whole, the surface of the oxide scale formed on the undoped alloy is mainly composed of Al_2_O_3_ and TiO_2_.

For the alloys doped with different rare earth elements, the surface of the oxide scale formed is also mainly composed of Al_2_O_3_ and TiO_2_, but the phases of HfO_2_, Al_2_Ti_7_O_15_, and HfTiO_4_ are not detected (Figure 4). The difference is that minor phases of Y_2_O_3_ and DyNbO_4_ are found on the scale surface for the alloys doped with Y and Dy, respectively. For the alloys doped with La, La-rich oxides are not detected on the surface of the oxide scales. The surface morphologies of the oxide scales also vary with the doping elements. Compared with the undoped alloy, the shapes of the TiO_2_ phase (the bright areas are shown in Figure 3) become rod-like from the original patch-like shape for the Y-doped alloy, while the TiO_2_ phase shows both rod-like and angularly patch-like shapes for the Dy- and La-doped alloy.

#### 3.2.2. The Cross-Sectional Microstructure of the NbSiTiAlHf Alloys After Oxidation

**Figure 5:**
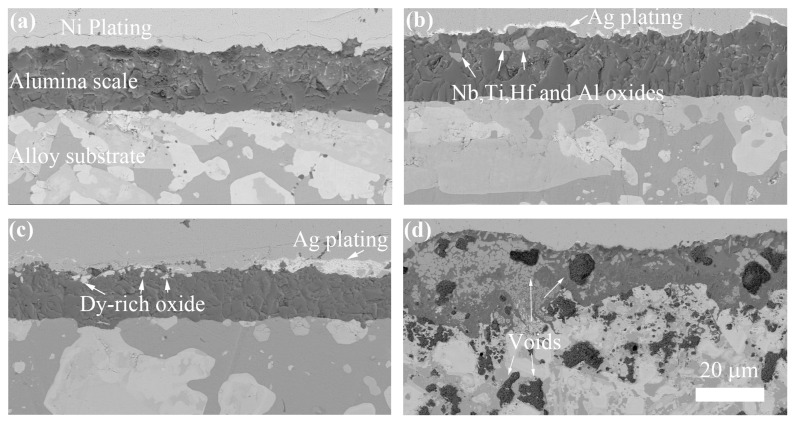
The cross-section microstructure of the NbSiTiAlHf alloys oxidized for 48 h at 1300 °C: (**a**) the undoped alloy; (**b**) doped with 0.5 at.% Y; (**c**) doped with 0.5 at.% Dy; and (**d**) doped with 0.5 at.% La.

**Figure 6:**
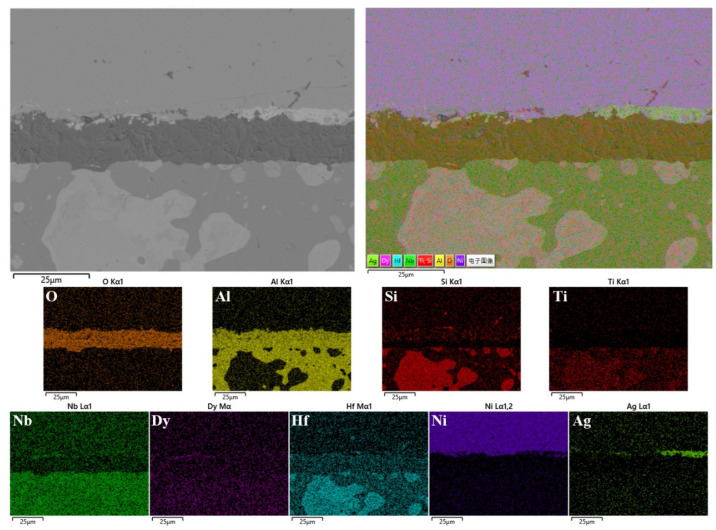
The cross-sectional images and corresponding elemental maps of the alloy doped with 0.5 at.% Dy, oxidized for 48 h at 1300 °C.

Figure 5 shows the cross-section microstructure of the NbSiTiAlHf alloys with and without rare earth elements oxidized for 48 h at 1300 °C: (a) undoped, (b) 0.5 at.% Y, (c) 0.5 at.% Dy. (d) 0.5 at.% La. The cross-sectional images and corresponding elemental maps of the alloy doped with 0.5 at.% Dy are shown in Figure 6. It can be seen that the thermally grown oxide scales are fully dense and continuous, and adhered well to the alloy substrates (except the 0.5 at.% La-doped alloys). Combined with the microstructure analysis of the scale surfaces, the whole scales are mainly composed of the major phases of Al_2_O_3_ and the minor phases of TiO_2_ (on the surface or in the upper layer of scales). The Al_2_O_3_ is most likely rich with small amounts of Si (on the surface or in the upper layer of scales, as shown in the Si map of Figure 6), and the TiO_2_ is rich with Nb and Hf, based on the EDS analysis. But, for the 0.5 at.% La-doped alloys, plenty of large voids with an average size of ~10 μm are observed in the oxide scale and near the scale/substrate interface. The major phases also changed into Al_2_O_3_ and TiO_2_ from Al_2_O_3_ compared with other alloys, since quantities of TiO_2_ are observed in the oxide scale.

### 3.3. The Oxidation Kinetics

**Figure 7:**
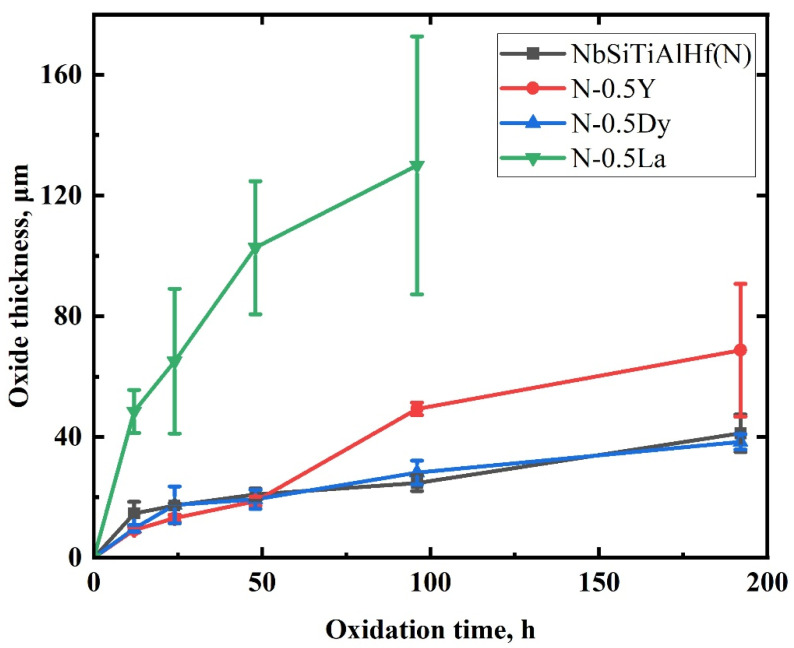
The average thickness of the oxide scale versus oxidation time on the NbSiTiAlHf alloys with and without rare earth elements, oxidized at 1300 °C.

Figure 7 shows the average thickness of the oxide scales versus oxidation time on the NbSiTiAlHf alloys with and without rare earth elements oxidized at 1300 °C (the oxide scale of the La-doped alloys spalls after 96 h oxidation at 1300 °C). Since the thickness of the oxide scale has sudden changes around 48 h oxidation duration for Y-doped alloys, the classic “Δh ^2^ = k_p_t” model should be replaced by a more general model that considers mixed (diffusion/reaction) control and a transient regime, as well as pure diffusion control. The values of the growth rate constant, $k_{p},$ are obtained through the generally parabolic equation [24,25]:

(1) $$t=A+B\Delta h+C\Delta h^{2}$$

(2) $$k_{p}=C^{-1}$$

where Δh is the average thickness of the oxide scale at time t, A, B, and C are the coefficients obtained from curve fitting. The evaluated k_p_ values of the different alloys are listed in Table 1.

It can be seen that the k_p_ of alloys with the addition of rare earth elements varies remarkably compared with that of the undoped alloys. Corresponding to the data of thickness mentioned above, the parabolic rate constant of 0.5 at.% Dy-doped alloys has the lowest value, where the k_p_ decreases by 20% compared with undoped alloys. But the k_p_ values increased by an order of magnitude for the Y and La-doped alloys, of which the k_p_ of the 0.5 at.% La-doped alloys has the highest value, showing a six-fold and nineteen-fold increase over that of the Y-doped alloys and the undoped alloys, respectively.

### 3.4. The Growth of the Oxide Scales

The calculations of the standard Gibbs free energy are of the oxide formation $\Delta G^{0}$ per mole O_2_ at the temperature of 1300 °C [26,27,28]. The Gibbs energies of the oxides $\Delta G^{0}$ of the selected oxides are arranged below:

$${\Delta G}_{Y_{2}O_{3}}^{0}<{\Delta G}_{{Dy}_{2}O_{3}}^{0}<{\Delta G}_{{La}_{2}O_{3}}^{0}<{\Delta G}_{HfO_{2}}^{0}<{\Delta G}_{{Al}_{2}O_{3}}^{0}<{\Delta G}_{TiO_{2}}^{0}<{\Delta G}_{SiO_{2}}^{0}<{\Delta G}_{{Nb}_{2}O_{5}}^{0}$$

According to the theory of thermodynamics, the lower $\Delta G^{0}$ implies the higher driving force of oxide formation and the higher thermodynamic stabilities of the selected oxides. Combined with XRD, the surface and cross-section analysis of the scales, the surface of the oxide scale formed consists mainly of Al_2_O_3_ and TiO_2_ for all of the alloys, although Al_2_O_3_ has higher thermodynamic stability compared to TiO_2_. This oxidation behavior could be attributed to the oxidation of Ti silicides and aluminides on the alloy surfaces at the early oxidation stage. Before the formation of a fully dense and continuous Al_2_O_3_ oxide scale, TiO_2_ grows together with Al_2_O_3_ due to its higher growth velocity compared with that of Al_2_O_3_ [26,29]. Other works also show that TiO_2_ is still formed even with the Al content up to 50 at.%, especially in γ-TiAl alloy [29,30]. Once a fully dense and continuous Al_2_O_3_ scale is formed, the growth of TiO_2_ could be suppressed, for the Ti-rich oxides can only be observed near the scale surface. The SiO_2_ could also be formed on the alloy surfaces at the early stage. Once a continuous scale is established, the growth of SiO_2_ could be suppressed by lower outward diffusion of Si due to its lower thermodynamic stability than TiO_2_ and Al_2_O_3_. The SiO_2_ could remain and dissolve in Al_2_O_3_, which is consistent with the observations that the Al_2_O_3_ is rich in Si on the surface or in the upper layer of scales (as shown on the Si map of Figure 6), where the results show that no SiO_2_ was detected by the XRD. In addition, the Al in silicides could suppress the formation of SiO_2_ beneath the alumina scale [15]. Reasonably, trace amounts of HfO_2_ and rare earth elements oxides should grow together with Al_2_O_3_, since all of the Y, Dy, La, and Hf have a higher driving force of oxide formation than Al. But HfO_2_ is only observed near the scale surface of undoped alloys, indicating that rare earth elements inhibit the formation of HfO_2_ on the scale surface. The formation of composite oxides Al_2_Ti_7_O_15_ and HfTiO_4_ is also suppressed for the doped alloys, probably owing to the doping elements slowing the outward diffusion of Ti, Hf, and Al by being segregated at the grain boundary of scales. The doping elements could dynamically segregate into the grain boundaries and then to the scale surfaces, driven by their higher oxygen affinity and the oxygen potential gradient across the metal–oxide–gas system [25,31]. Previous investigations reported that the NbSiTiAlHf alloys also formed thicker continuous Al_2_O_3_ scales at 1200 °C. But the oxidation products on the scale surface were more complex and consisted of Ti niobates, in addition to TiO_2_, SiO_2_, TiAl_2_O_5_, Nb_2_O_5_, HfO_2,_ and Al_2_O_3_. The difference could be attributed to the microstructural and compositional inhomogeneity [15,16].

It is well known that small amounts of rare elements such as Y and Dy could significantly reduce the growth kinetics of protective alumina- and chromia-based scales to improve the scale adhesion of alloys and coatings [18,25,31]. However, the growth rate constant, $k_{p},$ of alloys do not decrease with the addition of rare earth elements, and vary remarkably compared with those of the undoped alloys. For the Y-doped alloys, it can be seen that the significant increase in the oxidation rates of the Y-doped alloys after 48 h of oxidation (Figure 7) resulted in the higher $k_{p}$ compared with undoped and Dy-doped alloys, which means Y has a short-term effect on the oxidation behavior of the NbSiTiAlHf alloys, similarly observed in the FeCrAl and NiAl alloys or coatings [18,31,32]. Since Y has a higher affinity with oxygen and a smaller cation size, it can dynamically segregate into the grain boundaries and then to the scale surfaces earlier than Dy, reducing the growth rate of scales by the retarding effect at earlier times (Figure 7). Once yttrium is consumed, the slowing effect is weakened after the concentration of Y decreases along the oxide grain boundaries towards the free surface, due to faster outward diffusion. In addition, the doping elements on grain boundaries can also reduce the grain size of the scales by suppressing grain growth with a solute-drag effect [31]. The oxidation rate could increase significantly and even higher than that of undoped alloys due to more diffusion channels of Al and O in the scales with finer grains. For the Dy-doped alloys, the value of $k_{p}$ is the lowest, indicating Dy can obviously reduce the oxidation rates of the NbSiTiAlHf alloys, probably due to rare-earth effects mentioned above.

It is reported that the La_2_O_3_ addition to a Fe-20Cr or Ni-20Cr matrix resulted in a low isothermal rate and excellent scale adhesion [33,34]. The addition of La could be expected to be beneficial in the NbSiTiAlHf alloys. Differently, the La-doped alloys exhibit very rapid oxidation with the formation of a less protective scale. No La-containing oxides are observed on the surface of the scales, which means no rare-earth effects exist due to the lack of La outward diffusion. La may be over-doped in the alloys and have an optimal concentration that produces beneficial effects. The amount of La(0.20 at.%) could still be excessive for the NiAl-based alloys [34]. The over-doping of La could lead to internal oxidation and faster oxidation rates [18]. The plenty of large voids in the oxide scale and near the scale/alloy interface could weaken the adhesion of the scales; the rapidly growing oxides could lead to higher growth stresses, which could then induce an earlier spallation of the scale, accounting for the scale of the La-doped alloys spalling after 96 h oxidation at 1300 °C. The voids are thought to form by a Kirkendall-type mechanism due to the different elemental diffusion rates, and the void in the scales may be associated with the different growth stress of Al_2_O_3_ and TiO_2_.

The oxidation behavior of other NbSi-based alloys doped with Y and Dy was also investigated in other works [27,28]. It was found that Dy could lead to a larger weight gain for NbSi-based alloys after oxidation at 1250 °C for 58 h, and a higher amount of Dy addition resulted in a larger weight gain, although no changes in the phase constitution and the microstructures compared with the base alloy [28]. Unlike the element of Dy, the Y addition up to 0.3 at.% could improve the oxidation resistance of the same Nb-Si-based alloys. But the over-doped Y (0.5 at.% addition) could deteriorate the oxidation resistance. These opposite results could be attributed to the porous, nonuniform, and discontinuous oxide scale that was grown on the Nb-Si-based alloys, which primarily consisted of Nb_2_O_5_, Ti_2_Nb_10_O_29_, TiNb_2_O_7_, Ti_0.4_Cr_0.3_Nb_0.3_O_2_, and glassy SiO_2_ [27], compromising the effect of Y and Dy on oxidation behavior.

## 4. Conclusions

In this study, the effect of the rare earth elements (Y, Dy, and La) on the isothermal oxidation of the alumina scale-forming NbSiTiAlHf alloys was investigated. The conclusions are summarized as follows:

(1) The microstructures of the NbSiTiAlHf alloys consisted of the phases of Nb and Ti silicides, mainly identified as Nb_3_Si_2_, Ti_5_Si_4_, Nb_3_Si, and Ti_5_Si_3_, and the phases of Nb and Ti aluminides, mainly identified as (Nb,Ti)Al_3_. The phases of the alloys doped with different rare earth elements are almost the same as the undoped specimens. All of the alloys still exhibit the microstructural and compositional inhomogeneity after being annealed in a vacuum at 1300 °C for 24 h.
(2) The whole scales are mainly composed of the major phases of Al_2_O_3_ and the minor phases of TiO_2_ (on the surface or in the upper layer of scales). The Al_2_O_3_ is most likely rich with small amounts of Si, whereas the TiO_2_ is rich with Nb and Hf. But, for the 0.5 at.% La-doped alloys, plenty of large voids with an average size of ~10 μm are observed in the oxide scale and near the scale/substrate interface. The major phases changed into Al_2_O_3_ and TiO_2_ from Al_2_O_3_ compared with other alloys. The other difference is that minor phases of Y_2_O_3_ and DyNbO_4_ are found on the surface or in the upper layer of scales for the alloys doped with Y and Dy, respectively. But, for the alloys doped with La, La-rich oxides are not detected on the scale surface.
(3) The addition of Dy could effectively reduce the growth rate of oxide scale on the NbSiTiAlHf alloys. The parabolic rate constant of 0.5 at.% Dy-doped alloys has the lowest value, where the value decreases by 20% compared with the undoped alloys. The addition of La could deteriorate the oxidation resistance of the NbSiTiAlHf alloys, for which the parabolic rate constant of the 0.5 at.% La-doped alloys has the highest value, showing a six-fold and nineteen-fold increase over that of the Y-doped alloys and the undoped alloys, respectively. The benefits of Y on the oxidation rates were short-term ones, since there was a significant increase in the oxidation rates of the Y-doped alloys after 48h of oxidation compared with the undoped and Dy-doped alloys.

## Figures and Tables

**Table 1:** The parabolic rate constants (k_p_) for the alloys doped with different rare earth elements.

Composition (at.%)	*K_p_* (cm^2^/s)	Time (h)
NbSiTiAlHf (N)	2.38 × 10^−11^	192
N-0.5Y	1.41 × 10^−10^	192
N-0.5Dy	1.87 × 10^−11^	192
N-0.5La	4.55 × 10^−10^	96

## Data Availability

The original contributions presented in this study are included in the article. Further inquiries can be directed to the corresponding author.
